# 以自身免疫性淋巴细胞增殖综合征为首发表现的家族性噬血细胞综合征1例

**DOI:** 10.3760/cma.j.cn121090-20251221-00607

**Published:** 2026-05

**Authors:** 豪 张, 瑞 吕, 玉娇 贾, 瑞雪 张, 刚 安

**Affiliations:** 1 中国医学科学院血液病医院（中国医学科学院血液学研究所），血液与健康全国重点实验室，国家血液系统疾病临床医学研究中心，细胞生态海河实验室，天津 300020 State Key Laboratory of Experimental Hematology, National Clinical Research Center for Blood Diseases, Haihe Laboratory of Cell Ecosystem, Institute of Hematology & Blood Diseases Hospital, Chinese Academy of Medical Sciences & Peking Union Medical College, Tianjin 300020, China; 2 天津医学健康研究院，天津 301600 Tianjin Institutes of Health Science, Tianjin 301600, China; 3 兰州大学第一医院血液科，兰州 730000 The First Hospital of Lanzhou University, Lanzhou 730000, China

患者，男，43岁。2021年4月1日因“消瘦、乏力、盗汗半年，发热10 d”首次就诊。查体：贫血貌，颈部双侧、腋窝及腹股沟区触及多个肿大淋巴结，脾大。血常规：WBC 2.84×10^9^/L，HGB 90 g/L，PLT 39×10^9^/L，淋巴细胞比例77.1％。生化：总蛋白87.1 g/L，白蛋白32.2 g/L，球蛋白54.9 g/L。免疫球蛋白（Ig）G 32.8 g/L，IgA 7.26 g/L，IgM 3.21 g/L。抗核抗体阳性（1∶320），C反应蛋白24.1 mg/L，类风湿因子112 IU/ml。直接抗球蛋白试验IgG1阳性。细胞因子：IL-10 13.3 pg/ml。排除EB病毒等感染、炎症及肿瘤。外周血淋巴细胞亚群：CD3^+^CD4⁻CD8⁻TCRαβ^+^细胞（双阴性T细胞）占淋巴细胞3.29％。右腹股沟淋巴结病理：淋巴结T区不典型增生。骨髓细胞学：三系减低，淋巴细胞比例明显增高，易见不典型淋巴细胞。骨髓病理：增生极度活跃（>90％），T细胞广泛增生伴纤维组织增生。骨髓流式细胞术：成熟淋巴细胞群61.66％，髓系原始细胞0.15％，CD117、CD38表达减弱；粒系比例减低，SSC减小；CD3^+^T淋巴细胞未见异常单克隆增生；T/NK细胞表型未见明显异常。骨髓染色体FISH：TP53及ATM基因未见异常。骨髓IGH、IGK、IGL、TCRβ、TCRγ、TCRδ重排阴性。骨髓染色体核型：46,XY[20]。骨髓二代测序检测：FAS基因c.676+2T>C突变阳性（2类，与疾病可能相关），突变频率为2.9％；UNC13D基因c.2588G>A突变阳性（3类，临床意义不明）。CT：颈部、胸部、上腹、盆腔多发小淋巴结，部分肿大；两肺下叶索条，两侧胸膜增厚；肝左叶异常强化影；肝、脾增大。患者入院后仍反复发热，经验性抗感染治疗无效，诊断为自身免疫性淋巴细胞增殖综合征。给予泼尼松70 mg/d（1 mg·kg^−1^·d^−1^）治疗，体温恢复正常，血象好转，出院口服泼尼松治疗，病情稳定，半年内减停。

2025年1月15日患者因间断发热、腰部疼痛再次入院。血常规：WBC 1.57×10^9^/L，HGB 105 g/L，PLT 64×10^9^/L，淋巴细胞比例70.7％。生化：总蛋白81.1 g/L，白蛋白37.7 g/L，球蛋白43.38 g/L。铁蛋白：76 ng/ml。IgG 22.9 g/L，IgA 5.77 g/L。呼吸道合胞病毒RNA阳性。骨髓细胞学：增生活跃，粒红比例减低，淋巴比例增高，易见不典型淋巴细胞，符合淋巴细胞增殖性疾病。骨髓病理：增生极度活跃，三系细胞可见，T细胞增生伴纤维组织增生。双阴性T细胞占淋巴细胞3.33％。骨髓流式细胞术：淋巴细胞比例增高，T细胞比例明显增高，表达CD45RO，其中双阴性T细胞占T细胞的6.97％，部分表达CD7，CD95表达率为97.19％。骨髓IGH、IGK、IGL、TCRβ、TCRγ、TCRδ重排阴性。复查骨髓二代测序检测到FAS基因c.676+2T>C突变，频率为3.22％（2类，与疾病可能相关），UNC13D基因c.2588G>A突变频率为100％（1类，为致病突变位点）。鉴于患者UNC13D c.2588G>A突变为纯合突变，对其亲属进行基因检测，其弟为纯合突变，子女为杂合突变。患者入院后仍发热，最高体温39.4 °C，抗感染治疗无效，并出现快速的血细胞减少，纤维蛋白原降低，甘油三酯、铁蛋白升高等，诊断为家族性噬血细胞综合征-3型，予噬血细胞综合征（HLH）-94方案化疗及积极对症支持治疗，期间合并HLH脑病可能，予大剂量激素及戈利昔替尼治疗，并继续抗感染、输血、预防癫痫发作等治疗，患者病情好转，复查HLH相关实验室检查结果逐渐恢复，嘱后续有条件时行无关供者造血干细胞移植。

